# Investigation of Halcyon multi‐leaf collimator model in Eclipse treatment planning system: A focus on the VMAT dose calculation with the Acuros XB algorithm

**DOI:** 10.1002/acm2.13519

**Published:** 2022-01-09

**Authors:** Ryohei Miyasaka, SangYong Cho, Takuya Hiraoka, Kohei Chiba, Toru Kawachi, Tetsurou Katayose, Yuhi Suda, Ryusuke Hara

**Affiliations:** ^1^ Department of Radiation Oncology Chiba Cancer Center Chuo‐ku Chiba Japan; ^2^ Department of Radiotherapy Tokyo Metropolitan Cancer and Infectious Diseases Center Komagome Hospital Bunkyo‐ku Tokyo Japan

**Keywords:** Acuros XB, DLG, Halcyon, leaf trailing, VMAT

## Abstract

**Purpose:**

The dual‐layer multi‐leaf collimator (MLC) in Halcyon involves further complexities in the dose calculation process, because the leaf‐tip transmission varies according to the leaf trailing pattern. For the volumetric modulated arc therapy (VMAT) treatment, the prescribed dose for the target volume can be sensitive to the leaf‐tip transmission change. This report evaluates the dosimetric consequence due to the uncertainty of the dual‐layer MLC model in Eclipse through the dose verifications for clinical VMAT. Additionally, the Halcyon leaf‐tip model is empirically adjusted for the VMAT dose calculation with the Acuros XB.

**Materials and methods:**

For this evaluation, an in‐house program that analyzes the leaf position in each layer was developed. Thirty‐two clinical VMAT plans were edited into three leaf sequences: dual layer (original), proximal single layer, or distal single layer. All leaf sequences were verified using Delta4 according to the dose difference (DD) and the global gamma index (GI). To improve the VMAT dose calculation accuracy, the dosimetric leaf gap (DLG) was adjusted to minimize the DD in single‐layer leaf sequences.

**Results:**

The mean of DD were −1.35%, −1.20%, and −1.34% in the dual‐layer, proximal single‐layer, and distal single‐layer leaf sequences, respectively. The changes in the mean of DD between leaf sequences were within 0.2%. However, the calculated doses differed from the measured doses by approximately 1% in all leaf sequences. The tuned DLG was increased by 0.8 mm from the original DLG in Eclipse. When the tuned DLG was used in the dose calculation, the mean of DD neared 0% and GI with a criterion of 2%/2 mm yielded a pass rate of more than 98%.

**Conclusion:**

No significant change was confirmed in the dose calculation accuracy between the leaf sequences. Therefore, it is suggested that the dosimetric consequence due to the leaf trailing was negligibly small in clinical VMAT plans. The DLG tuning for Halcyon can be useful for reducing the dose calculation uncertainties in Eclipse VMAT and required in the commissioning for Acuros XB.

## 1 INTRODUCTION

Halcyon (Varian Medical Systems, Palo Alto, CA, USA) is the latest O‐ring linear accelerator (linac) with a single 6‐MV flattening filter‐free beam, fast gantry rotation speed of four rotations per minute, and a high dose rate up to 8 Gy/min.^1^ Additionally, a jawless design and a dual‐layer multi‐leaf collimator (MLC) with stacked‐and‐staggered leaves are introduced in the field aperture. The dual‐layer MLC can significantly decrease the leakage dose by blocking the inter‐leaf gaps.^2^ These mechanical features are designed to specialize in a dynamic dose delivery, such as volumetric modulated arc therapy (VMAT). Halcyon based VMAT is capable of achieving an adequate quality consistent (equivalent or better) with other consolidated advanced linacs.^3^, ^4^, ^5^, ^6^ In addition, fast gantry rotation and high dose rate dose delivery have the potential to reduce the treatment time and make treatment more robust to intrafractional patient motion.^7^, ^8^

However, the dual‐layer MLC system involves further complexities in the dose calculation process, because the transmission around the rounded leaf‐tip varies according to the leaf trailing pattern using both leaf layers. When the leaf trailing distance is larger, the leaf‐tip transmission is comparable to a standard MLC using a single layer. On the other hand, as the trailing distance decreases, the leakage dose becomes close to the mono‐block collimator. Hernandez et al. called this variation of transmission, the “leaf‐trailing effect.”^9^ They reported that the MLC model implemented in the Eclipse (Varian Medical Systems, Palo Alto, CA, USA) treatment planning system (TPS) failed to model the leaf‐trailing effect, and thus the calculated dose was underestimated for trailing distances ≥1 mm or overestimated for trailing distances ≤1 mm.

For VMAT treatment, the prescribed dose for the target volume can be sensitive to the change of leaf‐tip transmission. The dosimetric leaf gap (DLG) is one of the parameters that simulate the leaf‐tip transmission. In Eclipse TPS, DLG is adopted to reduce the dose calculation uncertainty arising from a simple MLC model with straight leaf ends.^10^ Szpala et al. reported that a 3% error in the target dose was introduced by a 1‐mm increase in the DLG for the VMAT dose delivery.^11^ Middlebrook et al. recommended that the DLG entered in Eclipse be 0.8 mm larger than the measured DLG in order to reduce the uncertainty of VMAT dose calculation with the Varian Millennium MLC model.^12^ As a result, the agreement between the calculated dose distribution and the film measurement improved, and the pass rate of the gamma index (GI) with a criterion of 3%/1.5 mm increased to more than 95%. Vieillevigne et al. reported that the configuring Eclipse with measured DLG may lead to large discrepancies of nearly 5% between measurements and calculations in VMAT stereotactic plans with the Varian high‐definition MLC model.^13^ Their investigation found that increasing the measured DLG in Eclipse by 0.7–0.8 mm greatly reduced the discrepancies. Similarly, several authors concluded that the improvements of the VMAT dose calculation accuracy can be achieved through better modeling of the rounded leaf‐tip.^14^, ^15^

On the other hand, the dose calculation uncertainties caused by the latest dual‐layer MLC model for Halcyon have not yet been investigated in the VMAT treatment plans. Lim et al. reported that the systematic dose difference (DD) between calculations with Anisotropic Analytical Algorithm (AAA, version 15.6) and measurements was around 1% in 10 VMAT dose verifications for Halcyon.^16^ However, they did not discuss the source of this difference and noted that it is subject to ongoing investigation and communication with the vendors. In order to ensure accurate dose calculation, careful attention should be given to the MLC model used in the Eclipse TPS configuration. In this report, the dosimetric consequence due to the uncertainty of the DLG value on the dual‐layer MLC model in Eclipse is evaluated through the dose verifications for clinical VMAT plans. Furthermore, most previous reports evaluated the AAA for Halcyon and did not focus on the Acuros XB, for which another dose calculation algorithm is implemented in Eclipse. To improve the VMAT dose calculation accuracy for Halcyon, the empirically adjusted DLG is investigated for the Eclipse TPS and the Acuros XB algorithm.

## 2 MATERIALS AND METHODS

**FIGURE 1:**
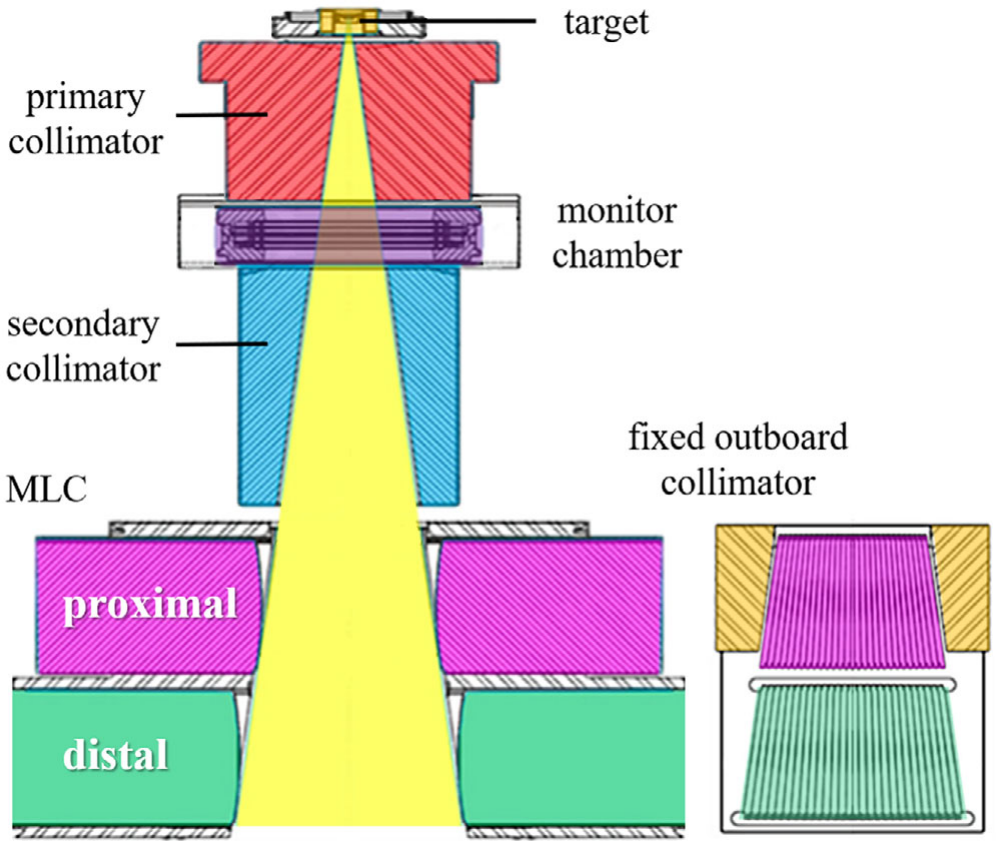
Schematic drawing of the components of the treatment head in Halcyon linac.^1^ As shown in left part, Halcyon is equipped with a primary collimator, a secondary collimator, and dual‐layer rounded leaf‐end multi‐leaf collimator (MLC) that is composed of 29 upper leaf pairs (proximal layer) and 28 lower leaf pairs (distal layer). The stacked‐and‐staggered leaves are arranged following a beam divergence, as shown in right bottom

As shown in Figure 1, Halcyon (version 3.0) is equipped with a primary collimator, a secondary collimator, and a stacked‐and‐staggered dual‐layer MLC that is composed of 29 upper leaf pairs (proximal layer) and 28 lower leaf pairs (distal layer). The leaves are designed with a rounded edge and arranged following a beam divergence. Each leaf is projected with a width of 10 mm at the isocenter plane, and thus dual‐layer MLC produced an effective resolution of 5.0 mm. The leaves are capable of traveling in a range from −14 cm to 14 cm at speeds up to 5 cm/s.

All treatment plans were created using Eclipse TPS (version 15.6). In addition, each plan was exported from TPS in a Digital Imaging and Communications in Medicine (DICOM) format and modified using an in‐house program (MATLAB R2021a, Mathworks, Natick, MA, USA) in this study, because the leaf motion cannot be manually assigned in Eclipse. The modified DICOM files were reimported into the TPS and calculated using Acuros XB (version 15.6) with a grid size of 2 mm.

### 2.1 Quantification of the leaf‐tip transmission

**FIGURE 2:**
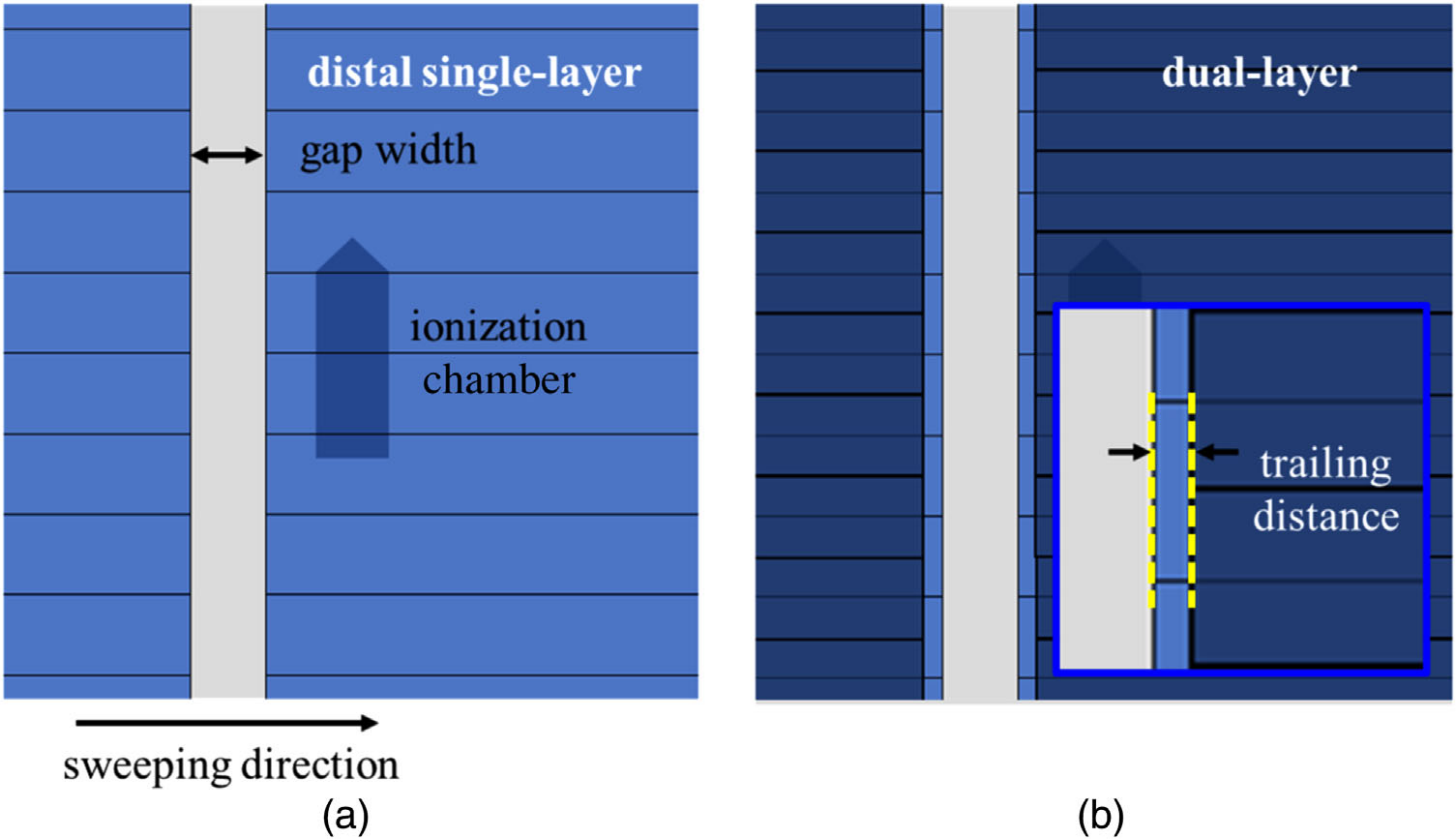
Beam's eye view of the sweeping gap field controlled by (a) the single‐layer and (b) the dual‐layer sequences. In the dual‐layer sequence, one leaf layer is trailed at a fixed distance (trailing distance) by another leaf layer

For quantification of the leaf‐tip transmission, the DLG in the proximal layer or distal layer MLC was evaluated. To determine the DLG at the beam axis, the dose ratio between the sweeping gap field and open field of 10 × 10 cm (*D*_ref_) was measured using an ionization chamber (30013, PTW, Freiburg, Germany) placed at a depth of 10 g/cm^2^ and a source–chamber distance of 100 cm. As shown in Figure 2a, the gap was set from widths of 2–20 mm in increments of 2 mm inside a 10 cm × 10 cm square field. The sweeping distance was set at 12 cm constantly in all sequences, and the leaf speed was maintained at 5 mm/s between all control points and all gap sizes using the in‐house program. The outputs of the sweeping gap field (*D*_g_) were corrected by the MLC transmission for each layer. The DLG was determined by the negative intercept of the linear fits between the dose ratio (*D*_g_/*D*_ref_) and sweeping gap width (*g*).

The leaf‐trailing effect was measured according to the “trailing sweeping gap test.”^9^ As shown in Figure 2b, the sweeping gap field was trailed by another layer at a fixed distance (*t*). The DLG was evaluated using the different trailing distances of 0, 0.5, 1, 2, 3, 4, 5, 10, or 20 mm. The inter‐leaf and intra‐leaf transmission were corrected according to the field size shielded by only a single layer, because the transmission through both layers was less than 0.01%.

In addition, the leaf motions and water phantom were simulated in the Eclipse and calculated. The DLG for Acuros XB dose calculation was also evaluated without and with the leaf trailing.

### 2.2 Verification of clinical VMAT treatment plans

The dosimetric consequence due to the leaf‐tip modeling uncertainty was evaluated through the clinical dose verifications for the VMAT treatment. In this study, 32 clinical VMAT plans were verified for the following target sizes: small (localized prostate cancer, *n* = 20) and large (whole pelvic region, *n* = 12). All plans were delivered with three arcs. To analyze the leaf sequences in the VMAT plans, the plan information was exported from the TPS in the DICOM format, and the gap widths and trailing distances were evaluated using the in‐house program.

All plans were calculated on a virtual cylindrical phantom, which modeled the commercial biplanar diode array device (Delta4 Phantom+ with Plastic Water DT, ScandiDos Inc., Ashland, VA, USA). The phantom density was assigned to 0.98 g/cm^3^ in accordance with our previous study.^17^ Each VMAT plan was read by Halcyon linac, delivered to Delta4, and measured as an absolute dose distribution. Before all measurements, the dose per monitor unit was obtained in accordance with the standard dosimetry protocol, and the daily output was corrected by the correction factor from the built‐in Delta4 software. The calculated and measured dose distributions were verified under the absolute dose mode according to DD and the global GI for the 2% dose difference criterion and the 2‐mm distance‐to‐agreement (2%/2 mm) criterion with the lower dose threshold of 10%. The absolute dose difference between any measured and calculated dose point pair was normalized using the maximum measured dose point (global normalization). The global normalization is recommended by an international guideline for IMRT measurement‐based dose verification.^18^

### 2.3 Influence of the leaf‐trailing effect in VMAT dose delivery

**FIGURE 3:**
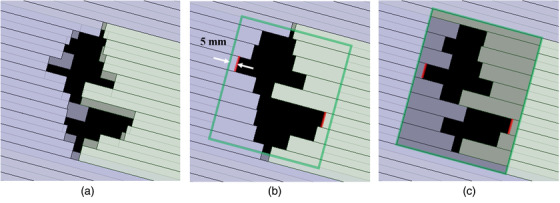
The volumetric modulated arc therapy treatment plan has three leaf sequences: (a) dual layer (clinical plan), (b) proximal single layer, or (c) distal single layer. In the single‐layer sequences, the green rectangle shows another layer's leaf edges, which are opened 5 mm away from the distal sides of one layer's leaves (red lines) similar to the conventional jaw tracking technique in C‐arm linacs

To evaluate the influence of the leaf‐trailing effect in VMAT dose delivery, each VMAT plan was exported from Eclipse in a DICOM format and modified using the in‐house program. As shown in Figure 3, (a) the dual‐layer leaf sequence (clinical plan) was edited into single‐layer leaf sequences with (b) the proximal MLC or (c) the distal MLC. In the single‐layer sequences, another layer's leaves were opened 5 mm away from the distal sides of one layer's leaves, similar to the conventional jaw tracking technique in C‐arm linacs. This modification can remove the leaf‐trailing effect and reduce the out‐of‐field dose. The single‐layer VMAT plans were imported into Eclipse and calculated using Acuros XB, then the calculated doses were verified in the same way as in Section 2.2.

### 2.4 DLG tuning for Halcyon

In order to improve the Halcyon MLC model in Eclipse TPS, the DLG was tuned specifically for the VMAT dose calculation with the Acuros XB algorithm. For the Halcyon, the original DLG in Eclipse is set to 0.1 mm in advance and cannot be changed by users. Consequently, each single‐layer leaf sequence was edited using the in‐house program and the leaf positions were opened in quantities of 0.2, 0.4, or 0.6 mm, respectively. The modified leaf sequences were reimported into Eclipse and recalculated with the same number of monitor unit using Acuros XB. Through this process, the DLG in the dose calculation was set to 0.5 mm (0.4 mm + original DLG in Eclipse of 0.1 mm), 0.9 mm (0.8 mm + 0.1 mm), or 1.3 mm (1.2 mm + 0.1 mm), respectively. These modified VMAT plans were not measured, because the purpose of the plan modification was only to simulate the calculations with another DLG entered in Eclipse. For the DLG tuning, the recalculated doses were compared to the measured doses in Section 2.3, and evaluated using the mean of DD. For each layer MLC, the DLG that minimized the mean of DD in single‐layer leaf sequences was considered as the empirically adjusted DLG (DLG_emp_).

In addition, the original VMAT plans were edited in DICOM format, and all leaf positions in each MLC layer were opened in a quantity of the “(DLG_emp_ – original DLG in Eclipse)/2” so that DLG_emp_ was used in Eclipse. Note that the DLG_emp_ assignment is limited to 0.2 mm intervals in this study, because the positional information for MLC was rounded to the nearest 10th in the Eclipse TPS. The modified plans were reimported into Eclipse and recalculated on a virtual cylindrical phantom using Acuros XB. The recalculated dose distributions were verified with respect to the Delta4 measurements in Section 2.2, and DD and GI were evaluated.

## 3 RESULTS

### 3.1 Quantification of the leaf‐tip transmission

**FIGURE 4:**
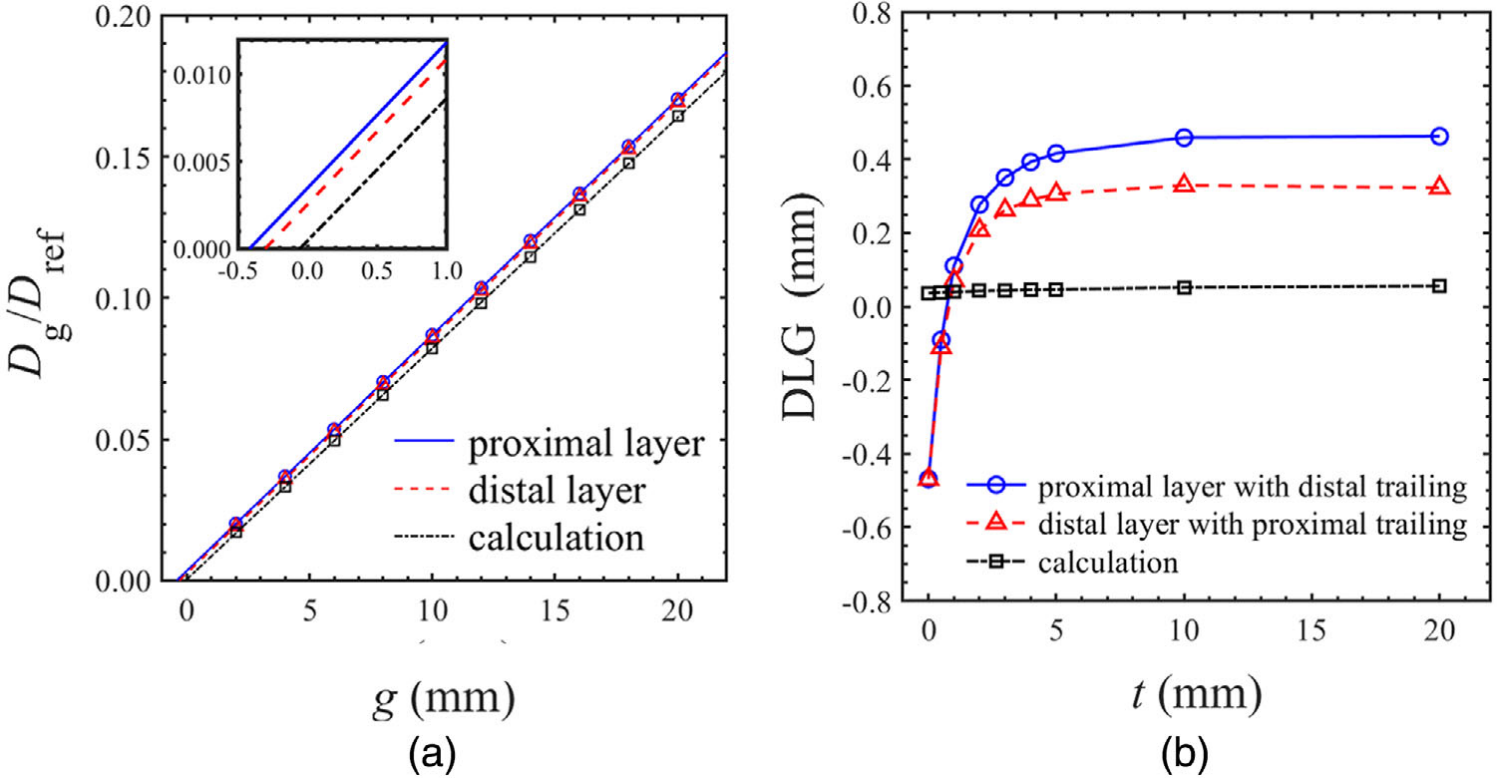
(a) The change of dose ratio with respect to sweeping gap width for each layer. The effective multi‐leaf collimator (MLC) transmission is subtracted from the dose ratio. Blue circles show the measurement using the proximal layer gaps, red triangles show the measurement using the distal layer gaps. The calculated dose ratio is constant for both layers. The lines are linear fits to the data using least‐squares regression, and the *x*‐axis negative intercept is defined as the dosimetric leaf gap (DLG). (b) The change of the DLG due to leaf trailing. The measured DLG drops sharply at trailing distances less than 5 mm

Figure 4a shows the change of dose ratio with respect to the sweeping gap width for each layer. The measured DLGs differed slightly between layers, 0.42 mm for the proximal layer and 0.32 mm for the distal layer, while calculated DLGs were 0.05 mm for both layers. In the trailing sweep gap test, the measured DLGs were comparable to the single layer's DLG at trailing distances from 5 to 20 mm, but dropped sharply at distances less than 5 mm, as shown in Figure 4b. The measured DLG value was −0.47 mm when the leaf projections coincided in both layers (trailing distance = 0 mm). However, no change in calculated DLG due to leaf trailing was obtained.

### 3.2 Verification of clinical VMAT treatment plans

Table 1 shows the gap width and the percentage of leaf trailing distance for each layer in the clinical VMAT plans. For a small target treatment, the median, 25th, and 75th percentile of gap width were 17.5 mm, 5.1 mm, and 30.1 mm, and the percentage of leaf trailing distances less than 5 mm was 59.8%.

**TABLE 1:** The median, 25th, and 75th percentile of gap width and the percentage of leaf trailing distance for each multi‐leaf collimator (MLC) layer in the clinical volumetric modulated arc therapy plans with different target sizes

				Percentage of the trailing distance (%)				
	MLC gap width (mm)			Proximal layer with distal layer trailing		Distal layer with proximal layer trailing		
Class of target size	Median	25th Percentile	75th Percentile	0 mm <* t* < 5 mm	5 mm ≤ *t*	0 mm <* t* < 5 mm	5 mm ≤ *t*	Complete overlap *t* = 0 mm
Small	17.5	5.1	30.1	19.4	17.3	16.1	22.9	24.3
Large	33.5	10.9	62.4	17.0	18.3	13.2	29.3	22.2

**FIGURE 5:**
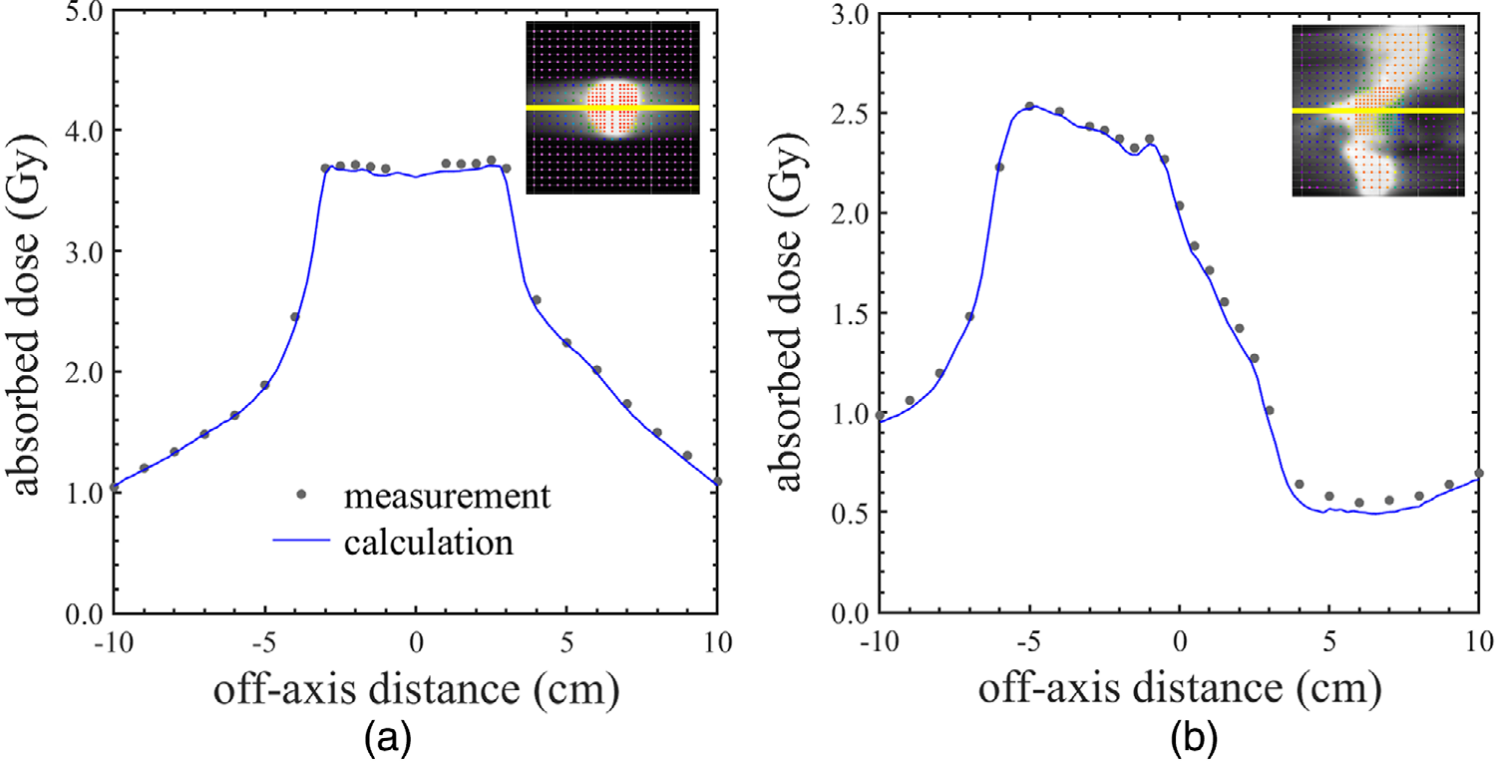
The measured and calculated dose profiles in Delta4 regarding the volumetric modulated arc therapy dose deliveries using the dual‐layer leaf sequence. These absolute dose verifications show (a) the coronal plane of localized prostate cancer, and (b) the sagittal plane of the whole pelvic region

Figure 5 shows the measured and calculated dose profiles in Delta4 regarding the VMAT dose deliveries. These absolute dose verifications show (a) the coronal plane of localized prostate cancer and (b) the sagittal plane of the whole pelvic region. The dose prescription was set to 3 Gy per fraction in localized prostate cancer and 2 Gy per fraction in the whole pelvic region through the VMAT optimization procedure. Note that the dose level and dose heterogeneity in the phantom measurement will be different to the original prescribed dose, because the phantom and patient body shapes are different. Table 2 shows the mean and standard deviation (SD) of DD and GI in clinical VMAT treatment plans for each target volume. For the gamma analysis, the pass rate was also evaluated. DD and GI between target sizes changed slightly even though the difference of MLC gap width was approximately double, as shown in Table 1. However, the calculated doses were underestimated with respect to the measurements. In both target classes, the mean of DD was around −1.3%, and the pass rate of GI with the 2%/2 mm criterion did not achieve 95%.

**TABLE 2:** The summary of the mean and SD of DD, and the GI with the criterion of 2%/2 mm in clinical volumetric modulated arc therapy treatment plans for each target volume

	DD (%)		GI (2%/2 mm)		
Class of target size	Mean	SD	Mean	SD	Pass rate (%)
Small	−1.31	1.85	0.43	0.30	94.93
Large	−1.41	1.33	0.51	0.32	92.30
Overall	−1.35	1.58	0.46	0.31	93.47

*Note*: For the gamma analysis, the pass rate of each criterion is evaluated.

### 3.3 Influence of the leaf‐trailing effect in VMAT dose delivery

Table 3 shows the mean and SD of DD and GI in the single‐layer leaf sequences. Compared to Table 2, the changes in the mean of DD between dual‐layer (original VMAT plan) and single‐layer leaf sequences were within 0.2%. Therefore, it is suggested that the dosimetric consequence due to the leaf trailing was insignificant during the VMAT dose delivery process. On the other hand, the systematic DD between the calculated and measured dose distributions was not improved even in the single‐layer leaf sequence. These results suggest that the uncertainty of the calculated dose is caused by the discrepancy of DLG in the dose calculation.

**TABLE 3:** The summary of the mean and SD of DD, and the GI with the criterion of 2%/2 mm in the single‐layer leaf sequences for each target volume

		DD (%)		GI (2%/2 mm)		
Class of target size	Leaf sequence	Mean	SD	Mean	SD	Pass rate (%)
Small	Proximal only	−1.18	1.75	0.40	0.25	98.17
	Distal only	−1.24	1.63	0.40	0.27	97.13
Large	Proximal only	−1.24	1.24	0.48	0.30	94.92
	Distal only	−1.49	1.25	0.53	0.32	91.52
Overall	Proximal only	−1.20	1.49	0.43	0.28	96.38
	Distal only	−1.34	1.44	0.45	0.31	94.04

### 3.4 DLG tuning for Halcyon

**FIGURE 6:**
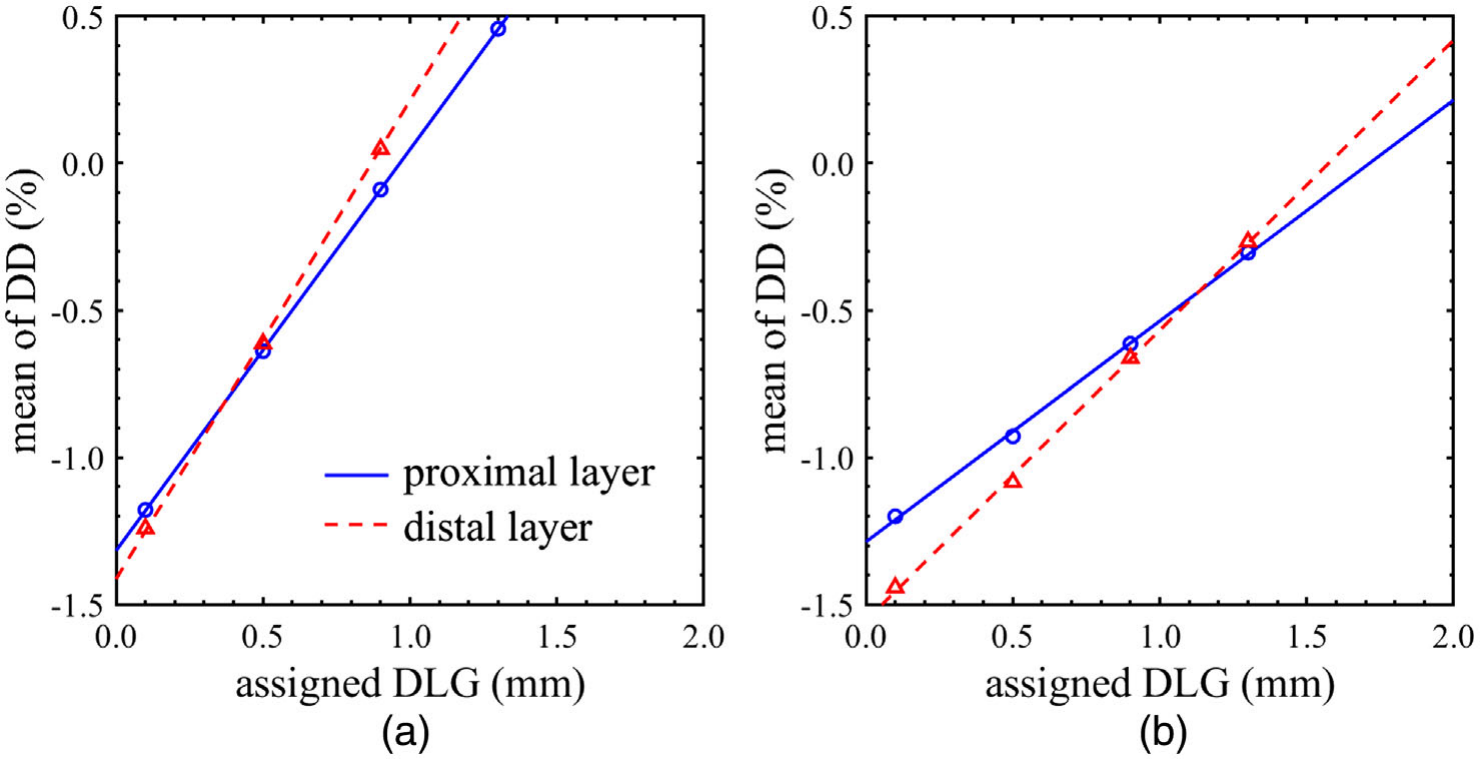
The mean of dose difference between calculations and measurements for the volumetric‐modulated arc therapy plans with proximal and the distal single‐layer leaf sequence. The dose differences obtained with various dosimetric leaf gap (DLG) values are shown for (a) small targets, and (b) large targets

As shown in Figure 6, the mean of DD changed linearly with various DLG values in the single‐layer VMAT plans for (a) the small and (b) the large targets. Therefore, the DLG_emp_ was determined by interpolating or extrapolating the results to identify the DLG that would produce a systematic DD close to 0%. Table 4 summarizes the DLG_emp_ in the proximal or the distal MLC layer for VMAT dose calculation. The DLG_emp_ was increased by around 0.8 mm for small targets and around 1.6 mm for large targets from the original DLG in Eclipse. In addition, the DLG_emp_ had around 0.1 mm difference between the MLC layers. From these results, in order to improve the dose calculation accuracy for VMAT plans with Halcyon, it is preferable that the DLG value in Eclipse can be edited individually for each MLC layer.

**TABLE 4:** The empirically adjusted dosimetric leaf gap (DLG_emp_) of the proximal or the distal multi‐leaf collimator (MLC) in volumetric modulated arc therapy dose calculation for small or large targets

	DLG_emp_ (mm)	
Class of target size	Proximal layer	Distal layer
Small	0.97	0.87
Large	1.71	1.57

**FIGURE 7:**
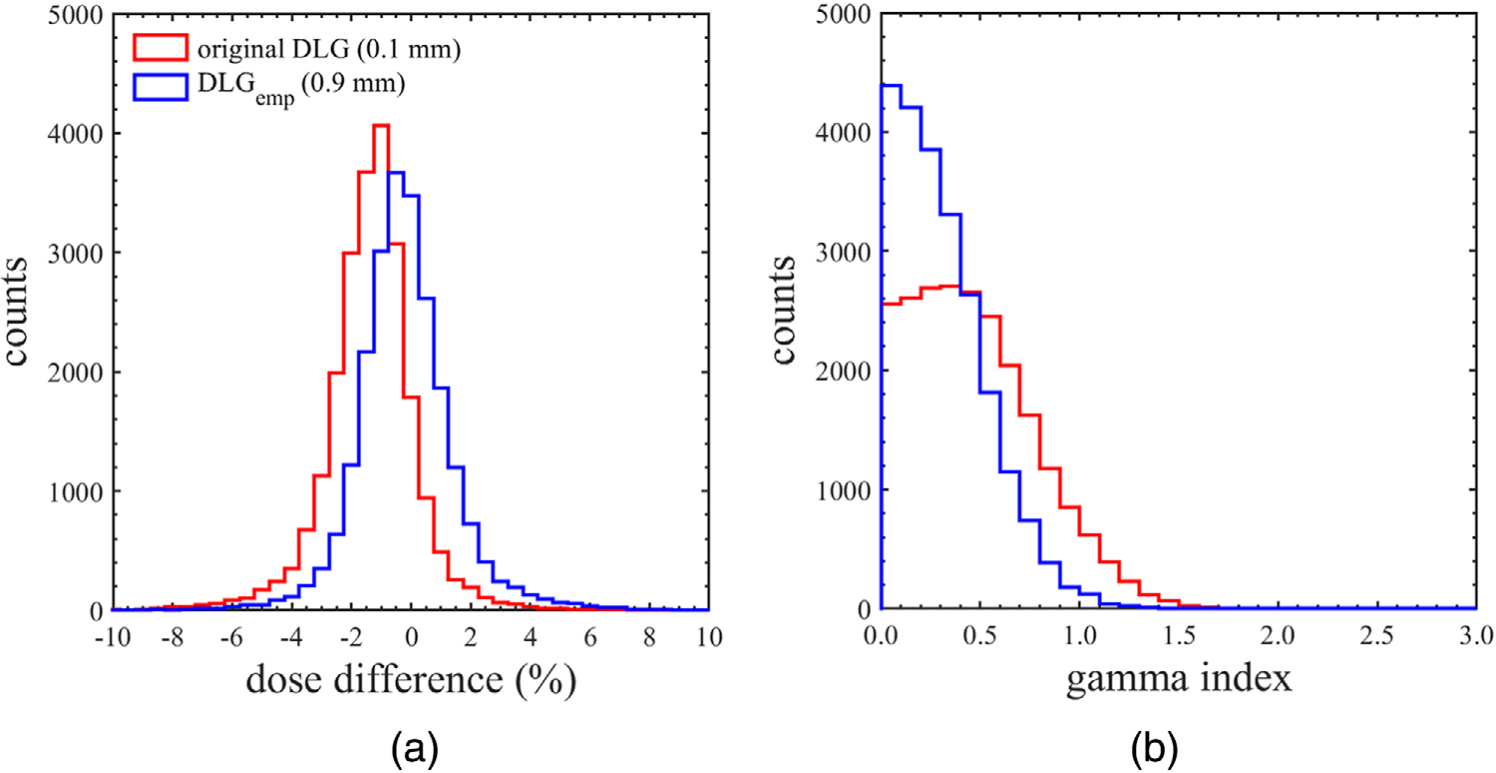
The histograms of (a) DD and (b) GI with the criterion of 2%/2 mm calculated with different dosimetric leaf gaps (DLGs; the original DLG of 0.1 mm and the DLG_emp_ of 0.9 mm) for the clinical volumetric modulated arc therapy (VMAT) plans. These histograms are plotted as a sum of VMAT dose verifications, not plan by plan

Table 5 summarizes the dose verifications in the clinical VMAT plans recalculated with the DLG_emp_. Compared to Table 2, the GI decreased and the mean of DD neared 0% in all plans when the DLG_emp_ for small targets was used in the VMAT dose calculation process. In addition, the recalculated dose distributions yielded a GI pass rate of more than 98% for the 2%/2 mm criterion. On the other hand, the DLG tuned for the large targets made DD and GI worse than the original DLG in overall dose verification. Comparing Figure 6a,b, the change in calculated dose with respect to the assigned DLG value is smaller in the VMAT plans for large targets. Therefore, it should be noted that the DLG_emp_ for the large targets was not appropriate for the small targets. In this study, the DLG_emp_ for the clinical VMAT plans was 0.9 mm, and the residual DD in large targets (about −0.5%) could be considered a good compromise. Figure 7 shows the histograms of the (a) DD and (b) GI in the clinical VMAT plans calculated with the original DLG and the DLG_emp_. The purpose of this investigation is to evaluate the systematic discrepancies due to the DLG entered in Eclipse. Therefore, these histograms were plotted as a sum of verifications, not plan by plan. As shown in Figure 7, it was found that increasing the original DLG in Eclipse by 0.8 mm significantly reduced the systematic discrepancies between the measured and the calculated doses in the clinical VMAT plans.

**TABLE 5:** The summary of the dose verifications for the clinical volumetric modulated arc therapy plans recalculated with the DLG_emp_ tuned for the small targets or the large targets

Assigned DLG in proximal‐layer MLC /distal‐layer MLC (mm)	Class of target size	DD (%)		GI (2%/2 mm)		
		Mean	SD	Mean	SD	Pass rate (%)
0.9/0.9 (tuned for the small targets)	Small	0.23	1.88	0.28	0.21	99.37
	Large	−0.52	1.37	0.34	0.24	98.95
	Overall	−0.05	1.66	0.30	0.23	99.13
1.7/1.5 (tuned for large targets)	Small	1.54	2.11	0.48	0.40	87.11
	Large	0.23	1.47	0.32	0.24	98.26
	Overall	1.05	1.90	0.42	0.33	93.32

*Note*: Empirically adjusted dosimetric leaf gap (DLG_emp_) assignment is limited to 0.2 mm intervals because the positional information for multi‐leaf collimator (MLC) was rounded to the nearest 10th in the Eclipse TPS.

## 4 DISCUSSION

To evaluate the dosimetric consequence due to the leaf‐tip modeling uncertainty, the verifications between the dose calculation and measurement were performed through the sweeping gap fields and VMAT dose deliveries. In the sweeping gap fields, DLGs were varied according to the inter‐layer or the leaf tracking distance. The previous study reported that the mean of DLG in five Halcyon linacs was 0.44 mm for the proximal layer (range 0.36 mm–0.48 mm) and 0.28 mm for the distal layer (range 0.12 mm–0.38 mm).^9^ It is clear that the leaf‐tip transmission in this study was within an acceptable level, because the measured DLGs showed good agreement with the international comparisons. Similarly, decreasing the DLG due to leaf trailing also reproduced their report.

Hernandez et al. reported that the relative differences between the calculated and measured doses in the leaf trailing condition ranged from −8% to 10% for the 5 mm gap, ±5% for the 10‐mm gap, and from −3% to 1% for the 20‐mm gap.^9^ The difference between the measured and calculated doses increases with narrower gap widths and shorter trailing distances. The important point to note is that these dose uncertainties were estimated in the simple slit fields. In the clinical VMAT plans, no significant change was confirmed in the dose calculation accuracy between the dual‐layer and the single‐layer leaf sequences even if the percentage of leaf trailing distances less than 5 mm is close to 60% in a small target treatment. Considering these results, it is suggested that the dosimetric consequence due to the leaf‐trailing effect was negligibly small in the standard VMAT treatment. However, the calculated doses had a systematic difference around 1% with respect to the measurements in dual‐layer and single‐layer leaf sequences.

In previous studies evaluating the VMAT plans with Halcyon, several authors verified only the AAA dose calculation and did not focus on the Acuros XB. The reason is that Acuros XB for Halcyon was recently released in Eclipse. Lim et al. reported that the dose discrepancy of the AAA (version 15.6) was −1.28% ± 0.80% (stated values are mean ± SD) for 10 VMAT dose verifications.^16^ The mean of DD in AAA was similar to this study. The comparison of dose calculation accuracy between Acuros XB and AAA has not been investigated in previous studies. In the sweeping gap test, the calculated DLG from Acuros XB was 0.05 mm, regardless of the MLC layer or trailing distance. This result was only slightly smaller than the calculated DLG from AAA (0.13 mm ± 0.01 mm) in previous report.^9^ The dose calculation algorithms in Eclipse use the identical MLC model; therefore, calculated dose may contain the same systematic uncertainty. It should be noted that SD was smaller than our result, probably because the evaluation was limited to a point dose in their study. Tamura et al. reported that the pass rate of GI with the 2%/2 mm criterion was 97.82% ± 2.61% for prostate cancer and 96.27% ± 2.13% for the head and neck region.^19^ Their report was slightly different from the GI pass rate in this study. The DLG in Eclipse for the Halcyon is set to 0.1 mm in advance and unified in all institutes, but it had a systematic discrepancy with respect to the measured DLG in each layer. It has been reported that the measured DLG differs between linacs within ±0.2 mm,^9^ so the delivered dose of the intensity‐modulated beam may vary depending on the user's Halcyon. Therefore, it is possible that tuning the DLG in TPS depending on each machine reduces the dose calculation uncertainties with Halcyon.

The DLG tuning in this study was performed manually in the DICOM format because Eclipse TPS does not allow users to change the beam model for Halcyon. This limitation made it difficult to reproduce the DLG_emp_ with a resolution of less than 0.1 mm in the VMAT dose calculation. As shown in Figure 6a, the dose variation per 1 mm of DLG is around 1.5% so that the recalculated dose may contain the uncertainty of 0.15%. In addition, the uncertainty of model of the MLC tongue‐and‐groove, transmission, penumbra, output factors for small field sizes, and off‐axis profiles was compensated only by tuning the DLG. Furthermore, it should be noted that the calculated dose is evaluated only by an IMRT verification device such as Delta4 in this study; therefore, the measured dose includes some uncertainties. The Delta4 measurement may have the dosimetric uncertainty of at least 0.5% even though daily output corrections are used, because the output is corrected for field sizes. These limitations can have an impact on the determination of optimal DLG.

In this study, the DLG in Eclipse was tuned in order to minimize the systematic discrepancies between measured and calculated doses in single‐layer leaf sequences. Increasing the DLG by 0.8 mm in the clinical VMAT plans significantly reduced these discrepancies in the small and large targets. Note that the DLG_emp_ specified for VMAT dose calculation with Acuros XB requires further tuning from the measured DLG. Vieillevigne et al. reported that the dose calculations in VMAT plans using the measured DLG were underestimated against the measurements, because the modeling of the MLC tongue‐and‐groove effect was insufficient in Eclipse TPS.^13^ Therefore, tuning the DLG is an effective method to compensate for the poor modeling of the tongue‐and‐groove. On the other hand, the leaf‐trailing effect cannot be modeled by tuning the DLG. Compared to Tables 2 and 3, the uncertainty of dose calculation due to the leaf trailing was small (around 0.2%) in the standard VMAT plans. However, it is suggested that the narrower the leaf gap width in the entire irradiation field, the greater the impact of the leaf trailing. In this case, a DLG_emp_ smaller than the DLG tuned by the single‐layer leaf sequences is anticipated from the perspective of Figure 4b. Therefore, modeling of the leaf‐trailing effect in Eclipse may be necessary, especially in the stereotactic VMAT plans for the smaller targets. Further investigation is necessary on the optimization of the DLG value for Halcyon.

## 5 CONCLUSIONS

In the dose verifications for clinical VMAT plans with Halcyon, no significant change was confirmed in the dose calculation accuracy between the dual‐layer and the single‐layer leaf sequences. Therefore, it is suggested that the dosimetric consequence due to the leaf trailing was insignificant in clinical VMAT plans. However, the original DLG in Eclipse may lead to some systematic discrepancies around ±1% in the dose calculation. In order to improve the Halcyon MLC model in Eclipse TPS, the DLG was tuned specifically for the VMAT dose calculation with the Acuros XB. The DLG that minimized the systematic discrepancies between measured and calculated doses in single‐layer leaf sequences was considered as the DLG_emp_ in this study. The DLG_emp_ was increased by around 0.8 mm from the original DLG in Eclipse. As shown in Figure 7, the dose verification results clearly improved in dual‐layer VMAT plans when the DLG_emp_ was used in the dose calculation process. This result suggests that adjusting the leaf‐tip model can be useful for reducing the dose calculation uncertainties and is necessary in the commissioning of Acuros XB for Halcyon. However, Eclipse TPS is not configured to individually assign the DLG value for each MLC layer, and the leaf‐trailing effect cannot be modeled. This compromise may make it difficult to tune the DLG value in the stereotactic VMAT plans for the smaller targets.

## CONFLICT OF INTEREST

The authors declare that there is no conflict of interest.

## AUTHOR CONTRIBUTIONS

Ryohei Miyasaka conceived the presented idea. All authors conceived and planned the work that led to the paper. Ryohei Miyasaka, SangYong Cho, Takuya Hiraoka, and Kohei Chiba performed the experiment. Ryohei Miyasaka and Yuhi Suda discussed the multi‐leaf collimator model uncertainty. Toru Kawachi, Tetsurou Katayose, and Ryusuke Hara supervised the study. Ryohei Miyasaka analyzed the data. All authors discussed the analysis method and results, wrote the paper, and approved the final version.
